# Associations between B Vitamins and Parkinson’s Disease

**DOI:** 10.3390/nu7095333

**Published:** 2015-08-27

**Authors:** Liang Shen

**Affiliations:** Shandong Provincial Research Center for Bioinformatic Engineering and Technique, School of Life Sciences, Shandong University of Technology, Zibo 255049, China; E-Mail: shen@sdut.edu.cn; Tel./Fax: +86-533-278-2220

**Keywords:** Parkinson’s disease, B vitamins, dietary intake, meta-analysis

## Abstract

B vitamins may correlate with Parkinson’s disease (PD) through regulating homocysteine level. However, there is no comprehensive assessment on the associations between PD and B vitamins. The present study was designed to perform a meta-analytic assessment of the associations between folate, vitamin B6, and vitamin B12 and PD, including the status of B vitamins in PD patients compared with controls, and associations of dietary intakes of B vitamins and risk of PD. A literature search using Medline database obtained 10 eligible studies included in the meta-analyses. Stata 12.0 statistical software was used to perform the meta-analysis. Pooled data revealed that there was no obvious difference in folate level between PD patients and healthy controls, and PD patients had lower level of vitamin B12 than controls. Available data suggested that higher dietary intake of vitamin B6 was associated with a decreased risk of PD (odds ratio (OR) = 0.65, 95% confidence intervals (CI) = (0.30, 1.01)), while no significant association was observed for dietary intake of folate and vitamin B12 and risk of PD. PD patients had lower level of vitamin B12 and similar level of folate compared with controls. Dietary intake of vitamin B6 exhibited preventive effect of developing PD based on the available data. As the number of included studies is limited, more studies are needed to confirm the findings and elucidate the underpinning underlying these associations.

## 1. Introduction

Parkinson’s disease (PD) is a geriatric neurodegenerative disorder with increasing global prevalence resulting in tremors, rigidity, and bradykinesia [1,2,3]. Although the mechanisms underlying the dopaminergic neurons degeneration in PD remain obscure, oxidative stress has been widely accepted to play a prominent role [4,5]. Homocysteine (Hcy) is a sulfur-containing metabolite generated in the essential amino acid methionine cycle. Hcy exhibits multiple neurotoxic effects involved in the pathogenesis of several neurodegenerative disorders, including dementia, Alzheimer’s disease and PD [6,7,8]. In recent years, many studies have investigated the associations between Hcy and PD and supported that patients with PD have increased levels of Hcy in comparison with age-matched healthy controls [9,10,11,12,13]. Increased levels of Hcy may lead to dopaminergic cell death in PD patients through neurotoxic effect [14,15,16] and thus, regulation of Hcy metabolism might decrease the risk of PD through decreasing plasma Hcy.

As methionine synthesis from Hcy requires B vitamins as cofactors, high B vitamins intake will decrease plasma Hcy level and may exert preventive effects of developing PD. However, to the best of our knowledge, there is no comprehensive assessment on the associations between PD and B vitamins. Thus, the present study was designed to comprehensively assess: (i) the B vitamins status in PD patients and controls; and (ii) efficacy of dietary intakes of B vitamins in lowering risk of PD, through meta-analyzing the available literatures. Levodopa is the most effective drug in the symptomatic management of PD and it was found that plasma Hcy levels are elevated in patients treated with levodopa. Thus, the meta-analyses of levodopa treated or untreated PD patients and controls were performed separately.

## 2. Methods

### 2.1. Literature Search

With the following search terms “Parkinson’s disease” and “B vitamins” or “folate” or “folic acid” or “vitamin B6” or “vitamin B12” or “cobalamin”, a literature search was performed in Medline database from inception until February 2015. To avoid missing potentially relevant studies by the search strategy, the reference lists of retrieved articles were also manually screened. A restriction to human studies and references published in English is imposed in the references selection. The review, opinion and editorial references as well as mechanistic studies were not considered. By reviewing titles, abstracts, and a full text of all citations identified with database searches, the potentially relevant references were identified on the basis of the following inclusion criteria.

### 2.2. Inclusion Criteria

Studies were included if they met the following criteria. The individual studies were examined and determined to be of sound science. For the meta-analysis of the B vitamins levels in PD patients, it was required that the included studies clearly provided the mean and standard deviation (SD) values of B vitamins levels for PD patients and matched controls. For the meta-analysis of the dietary intake of B vitamins and risk of PD, the odds ratios (ORs) or relative risks (RRs) or hazard ratios (HRs) with 95% confidence intervals (CI) of PD risk should be clearly provided.

### 2.3. Statistical Analysis

With prespecified data extraction tables, data was extracted from the included studies about the characteristics of study populations, B vitamin levels or PD risk and adjusting factors. The extracted data was used to obtain the corresponding standardized mean difference (SMD) and 95% CI of B vitamins levels in PD patients and controls, or OR and 95% CI of PD risk. Possible statistical heterogeneity between the eligible studies was assessed using the *I*^2^ statistic. The random-effect model was used to pool the SMD of B vitamins levels and OR of PD risk. All calculations were done with the Stata 12.0 statistical software (STATA Corp., College Station, TX, USA).

## 3. Results

**Figure 1:**
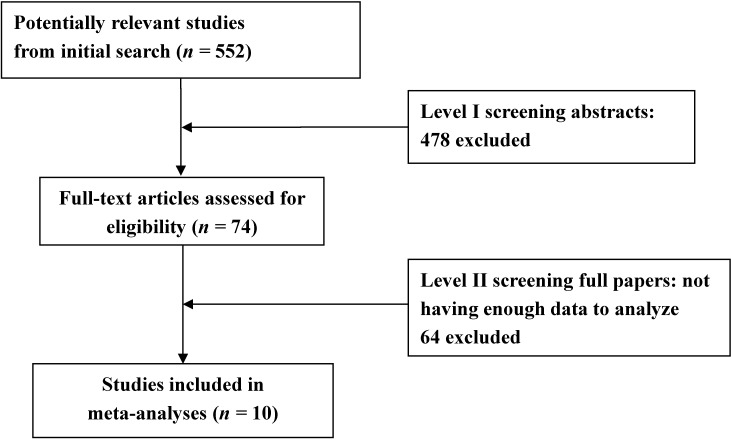
Search strategy for meta-analysis.

Figure 1 showed the literature selection and identification flow diagram. Our literature search resulted in 552 initial hits and 10 eligible studies finally included in the meta-analysis [17,18,19,20,21,22,23,24,25,26]. Seven studies including 10 study populations [17,18,19,20,21,22,23] provided data on folate and vitamin B12 levels in PD patients and controls, among which, 8 study populations were levodopa treated and 2 were levodopa untreated. 3 studies provided data on dietary intakes of B vitamins and PD risk [24,25,26].

**Figure 2:**
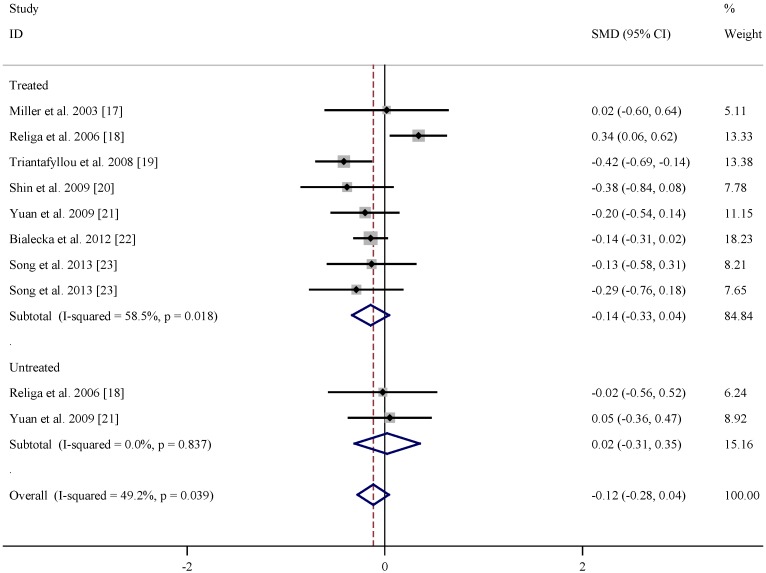
Pooled estimate of standardized mean difference (SMD) and 95% confidence interval (CI) of Parkinson’s disease (PD) and folate level in plasma.

**Figure 3:**
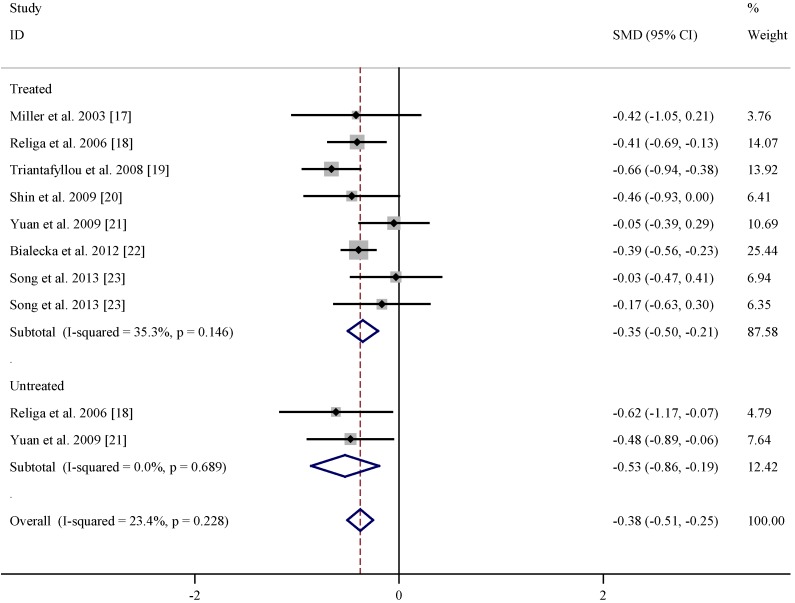
Pooled estimate of standardized mean difference (SMD) and 95% confidence interval (CI) of Parkinson’s disease (PD) and vitamin B12 level in plasma.

Table 1 listed the study and population characteristics of folate levels in PD patients and controls. According to the pooled data as shown in Figure 2, no obvious difference in folate level was found between levodopa treated or untreated patients and controls. Similar analysis on the basis of the extracted data in Table 2 indicated that vitamin B12 level was lower in both levodopa treated (summary SMD = −0.35, 95% CI = (−0.50, −0.21)) and untreated (summary SMD = −0.53, 95% CI = (−0.86, −0.19), Figure 3) PD patients than matched controls with low statistical heterogeneity. At present, there is not enough data of vitamin B6 levels to perform meta-analysis study.

**Figure 4:**
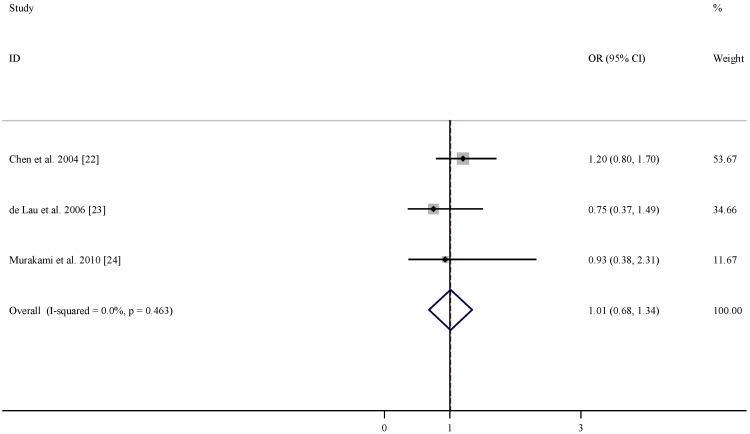
Forest plots of dietary intakes of folate and risk of Parkinson’s disease (PD).

**Figure 5:**
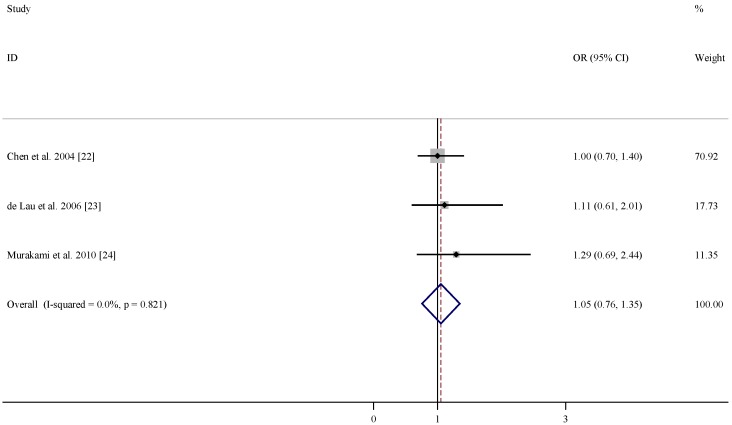
Forest plots of dietary intakes of vitamin B12 and risk of PD.

**Figure 6:**
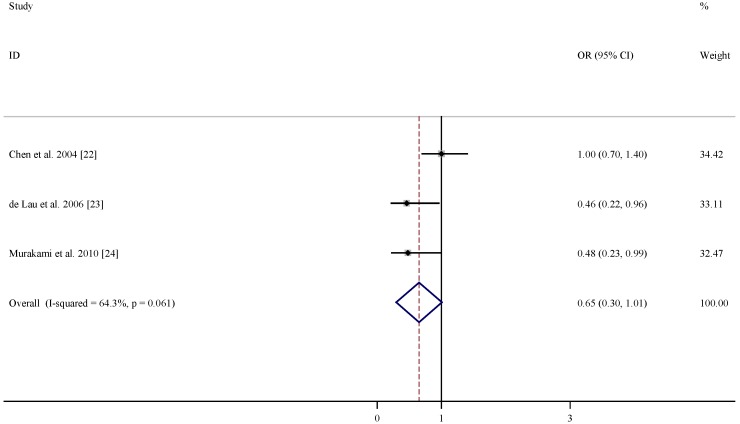
Forest plots of dietary intakes of vitamin B6 and risk of PD.

There were three studies included in the meta-analysis of the association between dietary intakes of folate, vitamin B6 and vitamin B12 and risk of PD (Table 3). According to the pooled OR, dietary intakes of folate and vitamin B12 were not associated with lower PD risks (OR = 1.01, 95% CI = (0.68, 1.34), Figure 4; and OR = 1.05, 95% CI = (0.76, 1.35), Figure 5). In comparison, dietary intake of vitamin B6 was related to decreased risk of developing PD (OR = 0.65, 95% CI = (0.30, 1.01), Figure 6) with moderate statistical heterogeneity.

**Table 1:** Characteristics of studies of folate levels (nmol/L) in levodopa treated and untreated Parkinson’s disease (PD) patients and controls.

References	Subgroup	Mean age (years)		*n*		Folate levels (nmol/L) (Mean ± SD)	
		PD	Control	PD	Control	PD	Control
Miller *et al.* 2003 [17]	Treated	64 ± 9	60 ± 10	20	20	12.8 ± 11.6	12.6 ± 10.8
Religa *et al.* 2006 [18]	Treated	70.5 ± 7.57	71.2 ± 6.0	99	100	20.87 ± 9.58	17.13 ± 12.21
Triantafyllou *et al.* 2008 [19]	Treated	70.1 ± 8.0	69.6 ± 8.1	111	93	9.92 ± 5.17	12.35 ± 6.57
Shin *et al.* 2009 [20]	Treated	63.5 ± 7.8	65.4 ± 7.8	33	41	19.26 ± 9.06	23.11 ± 10.87
Yuan *et al.* 2009 [21]	Treated	71.83 ± 10.34	69.95 ± 8.46	48	110	18.28 ± 10.8	20.37 ± 10.33
Białecka *et al.* 2012 [22]	Treated	64.4 ± 10.1	64.8 ± 9.6	320	254	20.16 ± 9.52	21.52 ± 9.28
Song *et al.* 2013 [23]	Treated	66.45 ± 6.60	66.23 ± 11.83	33	48	34.46 ± 25.69	38.54 ± 33.57
Song *et al.* 2013 [23]	Treated	70.50 ± 6.75	66.23 ± 11.83	28	48	29.47 ± 27.21	38.54 ± 33.57
Religa *et al.* 2006 [18]	Untreated	66.0 ± 7.11	71.2 ± 6.0	15	100	16.88 ± 6.39	17.13 ± 12.21
Yuan *et al.* 2009 [21]	Untreated	70.57 ± 9.09	69.95 ± 8.46	28	110	20.87 ± 8.11	20.37 ± 10.33

SD: standard deviation.

**Table 2:** Summary of studies regarding plasma vitamin B12 level (pmol/L) in in levodopa treated and untreated PD patients and controls.

References	Subgroup	Mean age (years)		*n*		Vitamin B12 levels (pmol/L) (Mean ± SD)	
		PD	Control	PD	Control	PD	Control
Miller *et al.* 2003 [17]	Treated	64 ± 9	60 ± 10	20	20	375 ± 167	464 ± 249
Religa *et al.* 2006 [18]	Treated	70.5 ± 7.57	71.2 ± 6.0	99	100	241.04 ± 129.04	17.13 ± 12.21
Triantafyllou *et al.* 2008 [19]	Treated	70.1 ± 8.0	69.6 ± 8.1	111	93	216.03 ± 90.5	283.54 ± 114.43
Shin *et al.* 2009 [20]	Treated	63.5 ± 7.8	65.4 ± 7.8	33	41	528.71 ± 299.70	651.93 ± 236.25
Yuan *et al.* 2009 [21]	Treated	71.83 ± 10.34	69.95 ± 8.46	48	110	354.76 ± 191.32	362.46 ± 135.82
Białecka *et al.* 2012 [22]	Treated	64.4 ± 10.1	64.8 ± 9.6	320	254	244.95 ± 104.77	295.12 ± 150.51
Song *et al.* 2013 [23]	Treated	66.45 ± 6.60	66.23 ± 11.83	33	48	473.61 ± 220.53	480.53 ± 239.45
Song *et al.* 2013 [23]	Treated	70.50 ± 6.75	66.23 ± 11.83	28	48	428.84 ± 265.56	470.61 ± 239.45
Religa *et al.* 2006 [18]	Untreated	66.0 ± 7.11	71.2 ± 6.0	15	100	201.42 ± 63.82	305.08 ± 178.03
Yuan *et al.* 2009 [21]	Untreated	70.57 ± 9.09	69.95 ± 8.46	28	110	299.95 ± 112.20	362.46 ± 135.82

PD: Parkinson’s disease; SD: standard deviation.

**Table 3:** Summary of studies regarding dietary intakes of folate, vitamin B6 and vitamin B12 and risk of PD.

References	Relative risk (95% CI) for folate	Relative risk (95% CI) for vitamin B6	Relative risk (95% CI) for vitamin B12	Adjustment
Chen *et al.* 2004 [22]	1.2 (0.8, 1.7)	1.0 (0.7, 1.4)	1.0 (0.7, 1.4)	age, smoking, total energy intake, alcohol consumption, caffeine intake, and lactose intake
De Lau *et al.* 2006 [23]	0.75 (0.37, 1.49)	0.46 (0.22, 0.96)	1.11 (0.61, 2.01)	age, sex, and total energy intake
Murakami *et al.* 2010 [24]	0.93 (0.38, 2.31)	0.48 (0.23, 0.99)	1.29 (0.69, 2.44)	age, sex, region, smoking, education, BMI, and dietary factors, including cholesterol, dietary glycaemic index, vitamin E , vitamin C, β-carotene, alcohol, caffeine and Fe, and intake of other B vitamins

PD: Parkinson’s disease; CI: confidence interval.

## 4. Discussion

In the present meta-analysis, the available data indicated no obvious difference in folate level between PD patients and controls, while vitamin B12 level was lower in PD patients than matched controls. Higher dietary intake of vitamin B6 may be associated with a decreased risk of PD, while there is no evidence to support the effects of folate and vitamin B12.

Homocysteine has been found to exhibit multiple neurotoxic effects and it is rational to infer that high dietary intakes of B vitamins may lower the risk of PD by decreasing plasma homocysteine levels. The present meta-analysis on the available studies suggested that there was no significant association between dietary intake of folate and vitamin B12 and risk of PD, while high dietary intakes of vitamin B6 was associated with decreased risk of PD. Thus, it is speculated that there may exist an alternative mechanism underlying the protective effects of vitamin B6 for PD. Oxidative stress has been widely accepted to play an important role in the pathogenesis of PD [4,5]. Besides its function as a cofactor, vitamin B6 is reported to possess antioxidant activity [27,28]. Pyridoxine is found to exhibit singlet oxygen quench capacity comparable with those of highly efficient antioxidants vitamins C and E [27]. It was demonstrated that vitamin B6 deficiency can lead to oxidative stress in rat liver and heart, while vitamin B6 supplementation can alleviate oxidative stress [29,30]. For instance, antioxidant activity of vitamin B6 can delay homocysteine-induced atherosclerosis in rats [31]. Moreover, it was reported that in stroke disease B vitamins supplementation may possess antioxidant and anti-inflammatory activities independent of the hypothesized homocysteine-lowering activity [32]. Thus, based on these evidences, it is proposed that besides regulating homocysteine levels the antioxidant potential of vitamin B6 may lower the risk of PD through inhibiting oxidative stress.

Several limitations of the present study warrant mention. First, the number of eligible studies is limited, especially for the analysis in the B vitamins levels in levodopa untreated PD patients. Second, the B vitamins dietary intakes are based on self-administered diet questionnaire, and the dosages varied in the included studies, which may potentially affect the results, and studies with control of people’s diet are recommended. Third, only studies published in English were included in the analysis.

To summarize, available data indicated that that PD patients had lower level of vitamin B12 and similar level of folate compared with controls. High dietary intake of vitamin B6 may be associated with a decreased risk of PD, while no obvious association was observed for dietary intake of folate and vitamin B12 and risk of PD. As the number of included studies is limited, more studies are warranted to confirm the findings and elucidate the mechanisms underlying these associations.
